# Lipid metabolism-related miRNAs with potential diagnostic roles in prostate cancer

**DOI:** 10.1186/s12944-023-01804-4

**Published:** 2023-03-14

**Authors:** Tianyuan Zhai, Meng Dou, Yubo Ma, Hong Wang, Fang Liu, Liandong Zhang, Tie Chong, Ziming Wang, Li Xue

**Affiliations:** 1 Department of Urology, The Second Affiliated Hospital of Xi’an Jiaotong University, Xi’an, 710004 Shaanxi China; 2 Department of Kidney Transplantation, Nephropathy Hospital, The First Affiliated Hospital of Xi’an Jiaotong University, Xi’an, 710061 Shaanxi China

**Keywords:** Prostate cancer, Lipid metabolism, microRNA, Diagnostic model, TF-miRNA‒mRNA network

## Abstract

**Background:**

Prostate cancer (PCa), the second most prevalent solid tumor among men worldwide, has caused greatly increasing mortality in PCa patients. The effects of lipid metabolism on tumor growth have been explored, but the mechanistic details of the association of lipid metabolism disorders with PCa remain largely elusive.

**Methods:**

The RNA sequencing data of the GSE45604 and The Cancer Genome Atlas-Prostate Adenocarcinoma (TCGA-PRAD) datasets were extracted from the Gene Expression Omnibus (GEO) and UCSC Xena databases, respectively. The Molecular Signatures Database (MSigDB) was utilized to identify lipid metabolism-related genes. The limma R package was used to identify differentially expressed lipid metabolism-related genes (DE-LMRGs) and differentially expressed microRNAs (DEMs). Moreover, least absolute shrinkage and selection operator (LASSO), extreme gradient boosting (XGBoost), and support vector machine-recursive feature elimination (SVM-RFE) were applied to select signature miRNAs and construct a lipid metabolism-related diagnostic model. The expression levels of selected differentially expressed lipid metabolism-related miRNAs (DE-LMRMs) in PCa and benign prostate hyperplasia (BPH) specimens were verified using quantitative real-time polymerase chain reaction (qRT‒PCR). Furthermore, a transcription factor (TF)-miRNA‒mRNA network was constructed. Eventually, Kaplan‒Meier (KM) curves were plotted to illustrate the associations between signature miRNA-related mRNAs and TFs and overall survival (OS) along with biochemical recurrence-free survival (BCR).

**Results:**

Forty-seven LMRMs were screened based on the correlation analysis of 29 DE-LMRGs and 56 DEMs, in which 27 LMRMs were stably expressed in the GSE45604 dataset. Subsequently, receiver operating characteristic (ROC) curves and machine learning methods were employed to develop a lipid metabolism-related diagnostic signature, which may be of diagnostic value for PCa patients. qRT‒PCR results showed that all seven key DE-LMRMs were differentially expressed between PCa and BPH tissues. Eventually, a TF-miRNA‒mRNA network was constructed.

**Conclusions:**

These results suggested that 7 key diagnostic miRNAs were closely related to PCa pathological processes and provided new targets for the diagnosis and treatment of PCa. Moreover, CLIC6 and SCNN1A linked to miR-200c-3p had good prognostic potential and provided valuable insights into the pathogenesis of PCa.

**Supplementary Information:**

The online version contains supplementary material available at 10.1186/s12944-023-01804-4.

## Introduction

Prostate cancer (PCa) is the most prevalent urological tumor [1]. Prostate-specific antigen (PSA), secreted by prostate epithelial cells, is used extensively for screening and diagnosing PCa. PSA screening significantly improves the PCa detection rate [2]. However, PSA is high in benign diseases, including prostatitis and BPH. Unnecessary prostate biopsies caused by the poor specificity of PSA lead to discomfort, bleeding, infection, and other complications [3]. Consequently, novel biomarkers for PCa diagnosis and risk stratification along with decision-making for prostate biopsy are needed.

Recent studies have shown that abnormal metabolic reprogramming, particularly in glycolysis [4], fatty acid metabolism [5], and cholesterol metabolism [6], can lead to many pathological changes, including inflammation and cancerization. Lipids provide nutrients for tumor cells and enhance their ability to adapt to the immune microenvironment. Intake of high-calorie foods and saturated animal fat is related to an increased incidence of PCa [7]. PCa cells show increased expression of several lipogenic enzymes [8]. MicroRNAs (miRNAs) are endogenous and short (22–25 nucleotides in length) noncoding RNAs [9] that degrade or inhibit mRNA translation by binding to the 3’ untranslated region of target mRNAs [10]. miRNAs act as regulatory factors for lipid metabolism and trafficking through associated enzymes and have been implicated in tumor cell proliferation and progression [11]. Numerous studies suggest that miRNAs can serve as diagnostic and prognostic biomarkers as well as therapeutic targets in PCa [12–17]. Nevertheless, the role of LMRMs in PCa and their mechanisms remain unclear.

The present study sought to identify key diagnostic miRNAs related to lipid metabolism and establish a potential TF-miRNA‒mRNA network using the RNA sequencing data of PCa, which might serve as clinically significant biomarkers and provide a reference for PCa diagnosis and prognosis.

## Materials and methods

### Data sources

The RNA sequencing data of the TCGA-PRAD dataset, including 499 PCa and 52 normal samples, were obtained from the UCSC Xena database (https://xenabrowser.net/datapages/), and 495 PCa samples with complete corresponding survival information were utilized for prognostic analysis. The GSE45604 dataset was acquired from the GEO database (https://www.ncbi.nlm.nih.gov/geo/query/acc.cgi?acc=GSE45604). MSigDB was utilized to extract lipid metabolism-related genes (LMRGs) [18].

### Identification of differentially expressed (DE)-LMRGs

DEGs between 499 PCa and 52 normal samples from the TCGA-PRAD dataset were selected by the limma R package (version 3.44.3) based on the threshold of |log_2_fold change (FC)|> 1 and *P* < 0.05. The expression levels of DEGs were displayed by heatmap and volcano plot via the pheatmap (version 4.1.0) and ggplot2 (version 3.3.2) R packages, respectively. DE-LMRGs were then identified by obtaining the intersection between LMRGs and DEGs, and DE-LMRG expression was assessed by the Wilcoxon test and visualized using a heatmap plotted using the pheatmap package (version 4.1.0).

### Specimens

PCa tissues (*n* = 10) and BPH tissues (*n* = 10) were collected from prostate biopsy specimens from patients admitted to The Second Affiliated Hospital of Xi’an Jiaotong University from December 2021 to May 2022. The Ethics Committee of the Xi’an Jiaotong University Health Science Center approved the research design on December 27, 2021 (protocol #: 2021–1700). Written consent was obtained from the patients before they donated their tissue samples.

### RNA extraction and qRT‒PCR

TRIzol® reagent (Ambion, MA, USA) was used to isolate total RNA from tissues. The M-MLV Kit (Accurate Biology, Changsha, China) was used to reverse transcribe (RT) total RNA (290 ng). The SYBR® Green qPCR Kit (Accurate Biology) was utilized for qPCR. U6 was the internal control. Fig. S 1 shows the U6 expression levels at the tissue and cellular levels. Table 1 shows the primer sequences. All reactions were repeated at least three times, and calculations were performed using the 2^−ΔΔCt^ method [19].

**Table 1:** Sequences of qRT‒PCR primers

Gene	Primer Sequences (5’-3’)
miR-148a-3p	RT: GTCGTATCCAGTGCAGGGTCCGAGGTATTCGCACTGGATACGACACAAAG
	F: CGTCAGTGCACTACAGAACTT
miR-187-3p	RT: GTCGTATCCAGTGCAGGGTCCGAGGTATTCGCACTGGATACGACCCGGCT
	F: CTCGTGTCTTGTGTTGCAGC
miR-200c-3p	RT: GTCGTATCCAGTGCAGGGTCCGAGGTATTCGCACTGGATACGACTCCATC
	F: CTAATACTGCCGGGTAATGAT
miR-3074-3p	RT: GTCGTATCCAGTGCAGGGTCCGAGGTATTCGCACTGGATACGACCGGTGC
	F: CGATATCAGCTCAGTAGGCA
miR-375-3p	RT: GTCGTATCCAGTGCAGGGTCCGAGGTATTCGCACTGGATACGACTCACGC
	F: CTTTGTTCGTTCGGCTCGC
miR-660-5p	RT: GTCGTATCCAGTGCAGGGTCCGAGGTATTCGCACTGGATACGACCAACTC
	F: CGCGTACCCATTGCATATCG
miR-93-3p	RT: GTCGTATCCAGTGCAGGGTCCGAGGTATTCGCACTGGATACGACCTACCT
	F: CAAAGTGCTGTTCGTGCAGG
universal primer	R: AGTGCAGGGTCCGAGGTATT
U6	F: CTCGCTTCGGCAGCACA
	R: AACGCTTCACGAATTTGCGT

*Abbreviations*: *F* forward, *RT* reverse transcribe, *R* reverse

### Identification of DE-lipid metabolism-related miRNAs (DE-LMRMs)

To identify LMRMs, DEMs between 52 normal and 499 PCa samples of the TCGA-PRAD dataset were first screened using the limma R package, considering |log_2_FC|> 1 and *P* < 0.05 as the screening criteria [20]. Next, DE-LMRMs were identified by performing Pearson's correlation analysis between DEMs and DE-LMRGs, and correlation < -0.3 and *P* < 0.05 were considered screening criteria. The overlapping DE-LMRMs and miRNAs expressed in samples from the GSE45604 dataset were used to identify stably expressed DE-LMRMs.

### Identification and validation of key diagnostic miRNAs

To assess the diagnostic utility of stably expressed DE-LMRMs, diagnostic miRNAs were identified from stably expressed DE-LMRMs (area under the ROC curve (AUC) > 0.8) by ROC curves constructed using the pROC package (version 1.17.0.1) [21] and were selected for further analysis. Thereafter, LASSO regression, support vector machine (SVM), and XGBoost algorithms were used to obtain candidate miRNAs using the glmnet package (version 4.0–2) [22], caret (version 6.0–92), and XGBoost (version 1.5.2.1), respectively. Overlapping miRNAs obtained from these three algorithms were defined as key diagnostic miRNAs. Furthermore, ROC curves were drawn to examine the predictive power of the diagnostic signature comprising the key diagnostic miRNAs and the individual key diagnostic miRNAs from the TCGA-PRAD dataset and GSE45604 dataset. Simultaneously, the levels of expression of key diagnostic miRNAs between different clinical subgroups were compared by Wilcoxon's tests (*P* < 0.05) and visualized using violin plots drawn using the ggplot2 (version 3.3.2) package to investigate the correlation between the key diagnostic miRNAs and different clinical characteristics.

### Establishment of a TF-miRNA‒mRNA network and prognostic analysis

To investigate the potential regulatory mechanism of key miRNAs, the StarBase and miRNET databases [23, 24] were utilized to predict potential binding sites of key diagnostic miRNAs and establish miRNA‒mRNA/TF-miRNA networks. The opposite expression patterns between miRNA and mRNA as well as TF were included to reduce the false-positive rate and exhibited through the Venn diagram. Then, the miRNA‒mRNA and TF-miRNA interactions were imported into Cytoscape software (version 4.0.2) [25] to generate the TF-miRNA‒mRNA network. Moreover, the correlations of PCa survival with gene expression of the factors involved in the network were analyzed based on the OS and BCR information of PCa cohorts, and the prognosis between the high- and low-expression groups was evaluated through KM survival curves using the survival package (version 3.1–12) [26].

## Results

### DE-LMRGs in PCa

**Fig. 1:**
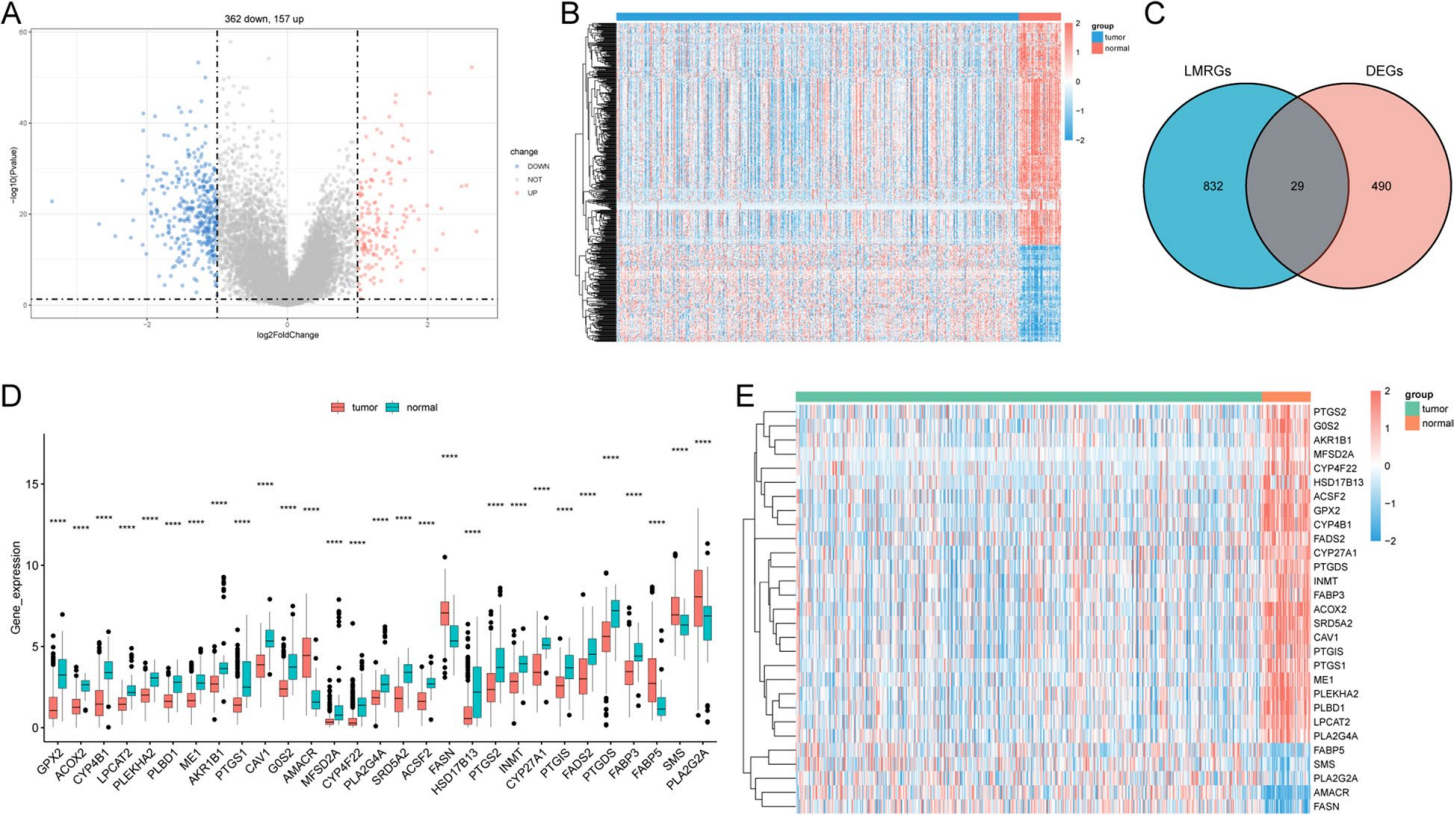
DE-LMRGs in PCa. **A** Volcano plot of DEGs in PCa versus normal tissues. **B** DEG-based cluster analysis of the PCa and normal samples. **C** Identification of DE-LMRGs between LMRGs and DEGs. **D** Expression of the 29 DE-LMRGs. ****P < 0.0001 vs. normal tissues. **E** Heatmap of DE-LMRG expression profiles

A total of 519 DEGs were derived from the TCGA-PRAD cohort, comprising 157 upregulated and 362 downregulated mRNAs (Fig. 1A and B). The intersection of lipid metabolism-related genes and LMRGs revealed 24 upregulated and 5 downregulated DE-LMRGs (Fig. 1C). The gene expression profiles of 29 genes are shown in Fig. 1D and Fig. 1E.

### DE-LMRMs in PCa

**Fig. 2:**
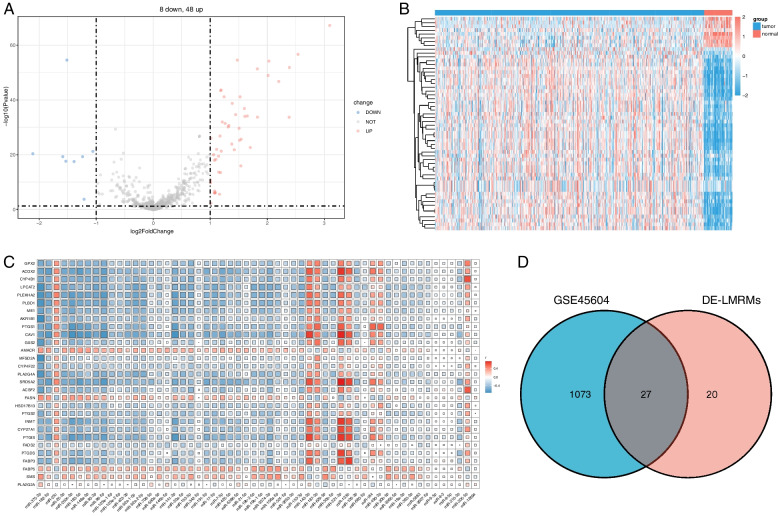
DE-LMRMs in PCa. **A** Volcano plot for DEMs between normal and PCa tissues. **B** Heatmap of the DEM expression profile. **C** Correlation between DEMs and DE-LMRGs. **D** Identification of stably expressed miRNAs between DE-LMRMs and miRNAs of the samples in the GSE45604 dataset

A total of 56 DEMs were found between 52 normal and 499 PCa samples, including 48 upregulated and 8 downregulated miRNAs (Fig. 2A and B). Subsequently, 47 DE-LMRMs closely related to lipid metabolism were identified based on the correlation between DEMs and DE-LMRGs (Fig. 2C), whereby 27 stably expressed miRNAs were utilized for further analysis (Fig. 2D).

### Identification and validation of key diagnostic miRNAs in PCa

**Fig. 3:**
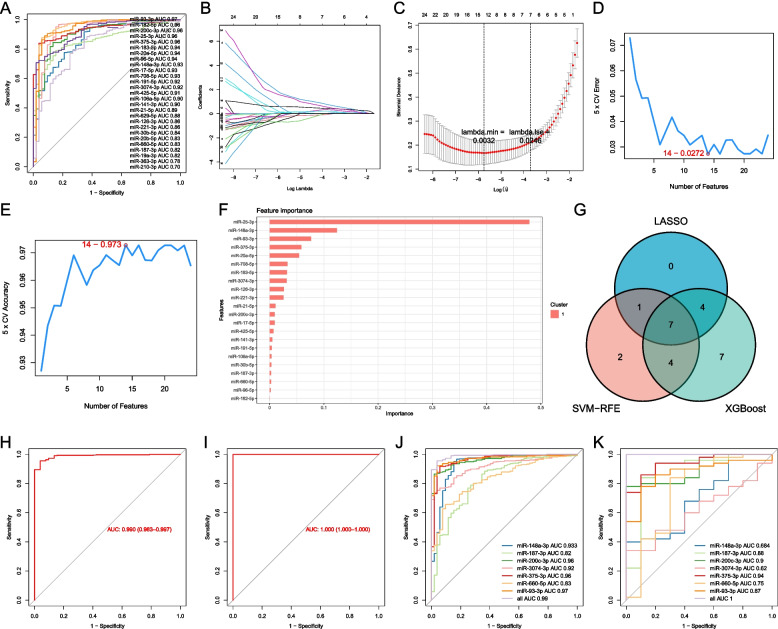
Identification and validation of key diagnostic miRNAs in PCa. **A** ROC curves for DE-LMRMs in distinguishing normal from PCa tissues. **B** The coefficient profile of 25 DE-LMRMs in LASSO regression. **C** The plot displays the cross-validation error according to the log of lambda value in the LASSO analysis. **D**, **E** Plots of generalization error and prediction accuracy versus the number of features in SVM, respectively. **F** Feature ranking based on the XGBoost machine learning algorithm. **G** Venn diagram for the diagnostic miRNAs screened by LASSO regression, SVM-RFE, and XGBoost algorithms. **H**, **I** ROC curves for the diagnostic signature in the training set and validation set, respectively. **J**, **K** ROC curves for each key diagnostic miRNA in the training and validation sets

**Fig. 4:**
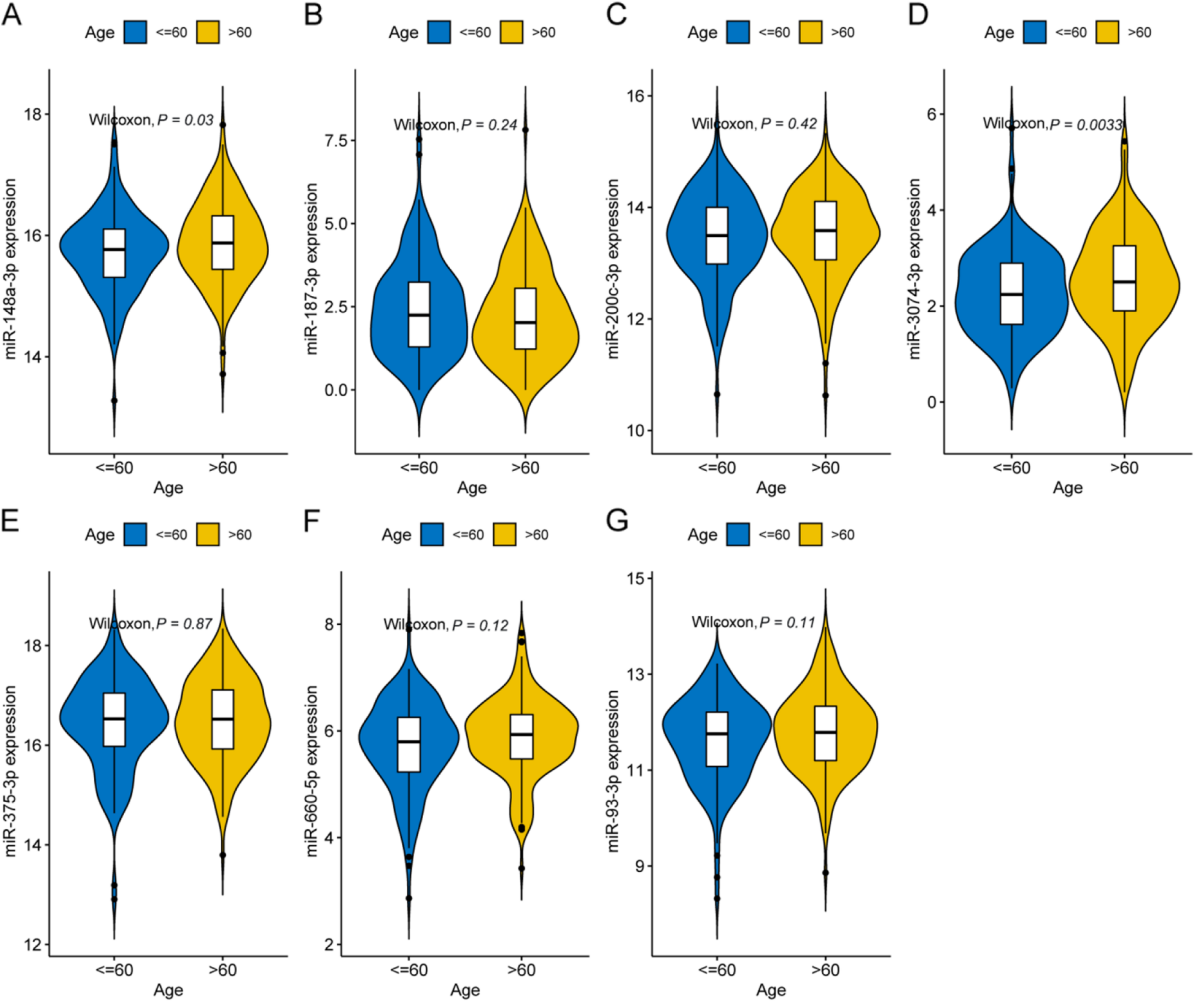
Differential analysis for signature miRNAs in different age groups

**Fig. 5:**
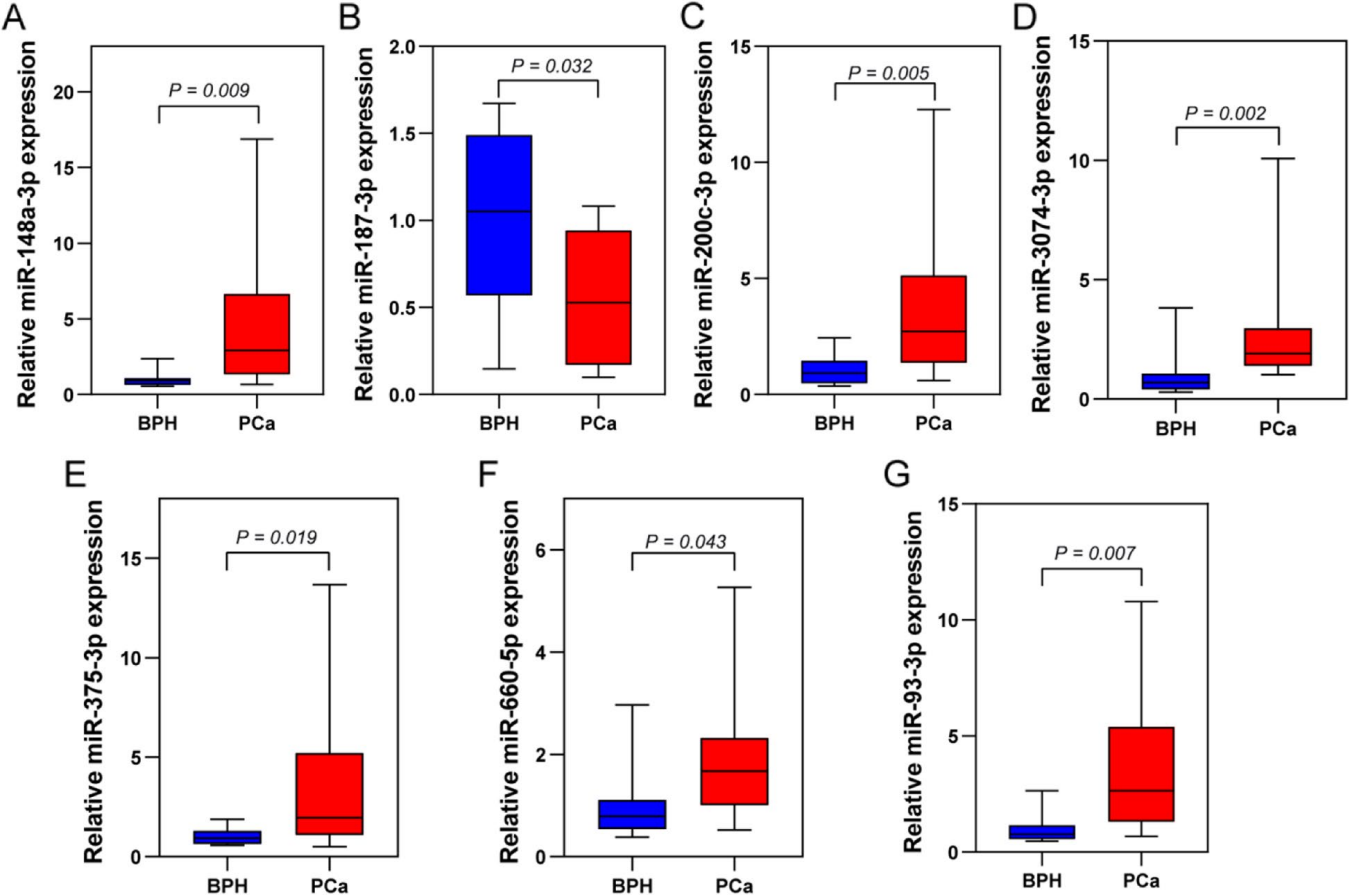
Expression levels of 7 key diagnostic miRNAs in PCa and BPH tissues

To screen key diagnostic miRNAs, an ROC analysis was performed to preliminarily narrow down the diagnostic miRNAs. As shown in Fig. 3A and Supplementary Table 1, 25 miRNAs showed a good distinguishing ability between normal and PCa samples and were regarded as diagnostic miRNAs. Subsequently, 7 key diagnostic miRNAs were screened by LASSO regression (Fig. 3B and C), SVM-RFE (Fig. 3D and E), and XGBoost (Fig. 3F) algorithms, including miR-148a-3p, miR-187-3p, miR-200c-3p, miR-3074-3p, miR-375-3p, miR-660-5p, and miR-93-3p (Fig. 3G). To validate the diagnostic signature's predictive ability, ROC curves based on the 7 key diagnostic miRNAs were plotted for the training set and validation set, suggesting that the AUCs were 0.99 and 1, respectively (Fig. 3H and I). Specifically, each key diagnostic miRNA also exhibited excellent distinguishing ability between the two datasets (Fig. 3J and K). Moreover, miR-3074-3p (*P* = 0.0033) and miR-148a-3p (*P* = 0.03) showed a high correlation with age (Fig. 4). qRT‒PCR data showed that the levels of expression of 7 key diagnostic miRNAs in PCa tissues differed significantly from those in BPH tissues (*P* < 0.05; Fig. 5).

### TF-miRNA‒mRNA network and prognostic analysis

**Fig. 6:**
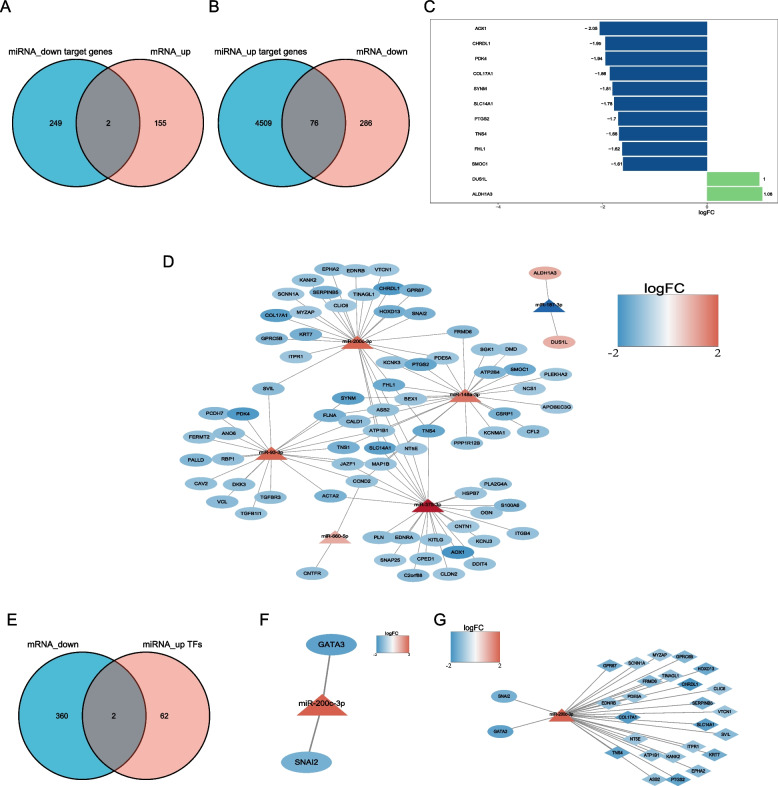
Establishment of the TF-miRNA‒mRNA regulatory network. **A** Venn diagram of upregulated mRNAs in PCa specimens and downregulated miRNA target genes. **B** Venn diagram of downregulated mRNAs in PCa and upregulated miRNA target genes. **C** The top 10 mRNAs in the miRNA‒mRNA network. **D** The miRNA‒mRNA network. **E** Venn diagram of downregulated mRNAs in PCa and TFs of upregulated miRNAs. **F** The miRNA-TF pairs. **G** The TF-miRNA‒mRNA network

**Fig. 7:**
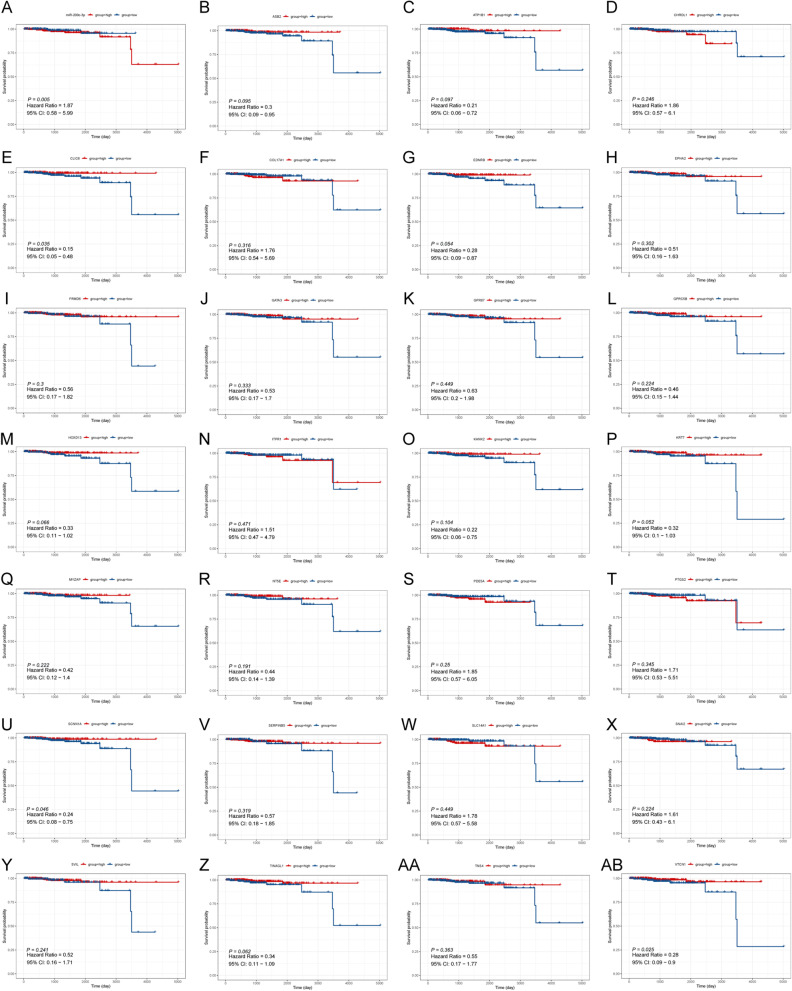
OS analysis based on TF-miRNA‒mRNA network-related genes

**Fig. 8:**
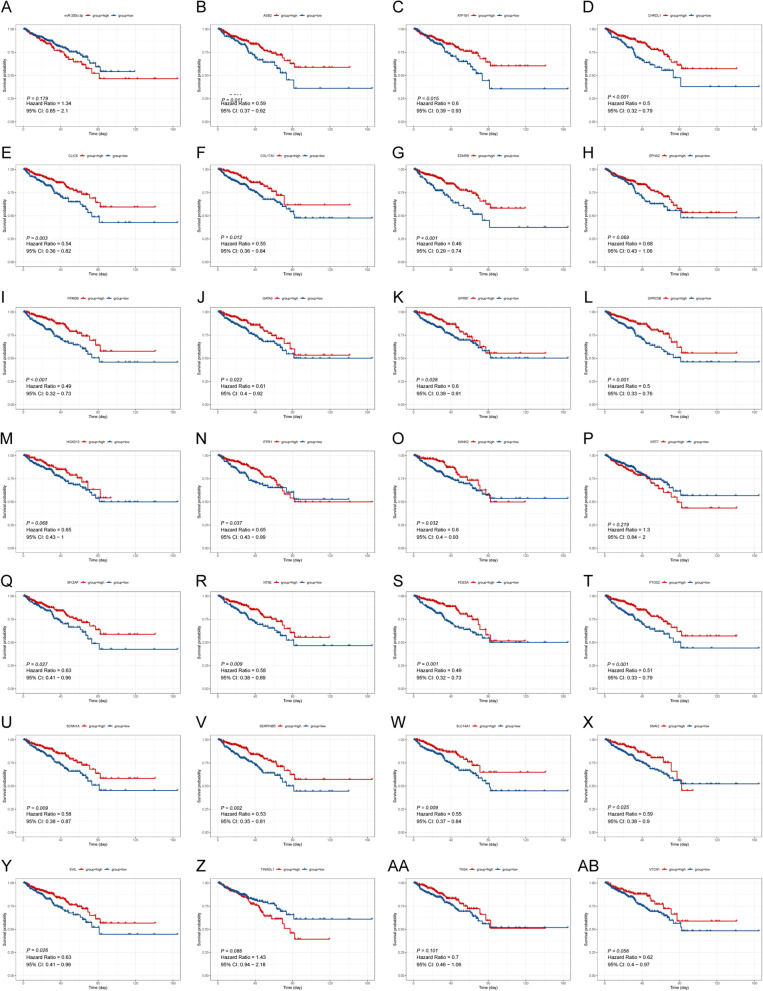
Biochemical recurrence-free survival analysis based on TF-miRNA‒mRNA network-related genes

Through the StarBase and miRNET databases, a total of 76 downregulated and two upregulated predicted mRNAs were considered key miRNA target sites and used to construct the miRNA‒mRNA regulatory network (Fig. 6A-D). Similarly, two TF binding sites of miR-200c-3p were predicted, namely, GATA3 and SANI2 (Fig. 6E-F). Following the integration of the obtained miRNA-TF (Fig. 6F) and miRNA‒mRNA networks (Fig. 6D), 25 targeted mRNAs as well as two TF binding sites of miR-200c-3p were used to construct the TF-miRNA‒mRNA network (Fig. 6G**)**. Of these, only 3 mRNAs (VTCN1, CLIC6, and SCNN1A) exhibited significant correlations with the OS of 495 PCa samples (*P* < 0.05) (Fig. 7). Simultaneously, 21 of 25 mRNAs (PDE5A, CLIC6, BDNRB, etc.) were confirmed to affect the BCR of PCa patients** (**Fig. 8). The results suggested that CLIC6 and SCNN1A may have good prognostic value for PCa and warrant further analysis.

## Discussion

Accumulating evidence has linked lipid metabolism disorders to the oncogenesis and development of several cancers, including bladder cancer [27], gastric cancer [28], and PCa [29]. PCa is characterized by increased fatty acid oxidation and de novo lipogenesis to satisfy the anabolic and energy demands of cancer cells [30]. Furthermore, hypercholesterolemia promotes the development of PCa and is a risk factor for progression toward castration-resistant PCa [6, 31]. Unfortunately, no studies on the role of LMRMs in PCa prognosis have been released. In contrast to previous methodological studies that directly investigated the relevance of miRNAs to diseases (e.g., breast cancer and lung cancer) by constructing similarity- or machine-learning-based models [32–35], the present study first identified DE-LMRMs by performing Pearson's correlation analysis between DEMs and DE-LMRGs and subsequently used three machine learning algorithms to finalize a diagnostic model for PCa consisting of seven signature miRNAs and constructed a lipid metabolism-related TF-miRNA‒mRNA network, providing novel targets for diagnosing and treating PCa.

Using ROC curves and three machine learning algorithms, along with qRT‒PCR verification, 7 key DE-LMRMs were identified, which may have great diagnostic value for PCa patients. Most of them are reportedly related to lipid metabolism and the development of PCa. For instance, miR-148-3p, as an important regulator of liver low-density lipoprotein receptor expression and lipoprotein metabolism, is upregulated in the serum of PCa patients [15, 36, 37]. miRNA-187-3p expression is significantly low in PCa tissues; its levels are lowered further in metastatic PCa patients [38]. miR-200c-3p has been reported to be positively associated with the development of nonalcoholic fatty liver disease (NAFLD) [39, 40]. High serum miR-200c-3p levels are reported in high-risk PCa patients, and low miR-200c-3p expression inhibits PCa cell proliferation [12, 41]. miR-375-3p is significantly upregulated in the serum of NAFLD patients [42]. Circulating and urinary miR-375-3p levels are markedly high in PCa patients [13, 43, 44]. Similarly, miR-660-5p is reportedly upregulated in the urine vesicles of PCa patients [45]. miR-93-3p promotes PCa cell invasiveness, and increased miR-93-3p levels are related to the progression and metastasis of PCa [14, 17]. The above findings are consistent with this study, strongly indicating that these LMRMs are promising diagnostic biomarkers for PCa. Interestingly, the present findings showed that miR-148a-3p and miR-3074-3p expression were positively correlated with the age of PCa patients.

Furthermore, a comprehensive study exploring both upstream transcription and downstream target regulation by LMRMs was conducted. A potential TF-miRNA‒mRNA regulatory network containing 1 miRNA, 25 mRNAs, and 2 TFs was established. SNAI2 and GATA3 may have a repressive role in miR-200c-3p transcription in PCa. The expression of SNAI2 has differential clinical significance across the stages of PCa. Silencing SNAI2 in PCa contributes to its high proliferation. However, metastatic tumors are characterized by high invasiveness and slow cellular proliferation, supported by the activation of SNAI2 [46]. GATA3, a zinc-binding TF, inhibits PCa progression and metastasis [16, 47]. Among the target genes of miR-200c-3p, high expression of CLIC6 and SCNN1A was related to a better prognosis in terms of OS and BCR in PCa patients, as reported here for the first time. However, CLIC6 has no reported significance in tumorigenesis and tumor progression to date. SCNN1A promotes ovarian and pancreatic cancer cell proliferation and migration and inhibits osteosarcoma growth [48, 49]. Nevertheless, the specific biological functions of CLIC6 and SCNN1A in PCa warrant further investigation.

### Comparisons with other studies and what does the current work add to the existing knowledge

When investigating the relationship between lipid metabolism and PCa, most previous studies focused on the role of LMRGs in the oncogenesis, progression, and prognosis of these patients or on the role of a specific miRNA in promoting or inhibiting the progression of PCa through lipogenic enzymes or regulating key TFs regulating lipid metabolism. However, the role of LMRMs in PCa and its related mechanisms remain unclear. For the first time, this study applied LASSO, SVM-RFE, and XGBoost algorithms to select signature miRNAs and constructed a lipid metabolism-related diagnostic model. In addition, a TF-miRNA‒mRNA network was established to investigate the regulatory mechanism of LMRM action in PCa.

### Study strengths and limitations

The study’s strength is that it investigated the role of LMRMs in PCa for the first time and identified seven hub genes with excellent diagnostic value in PCa. Furthermore, this study established a TF-miRNA‒mRNA network, providing a novel target for diagnosing and treating PCa. The flaws of this study are shown below. First, the diagnostic hub genes were detected only and validated by public datasets, and the diagnostic value needs to be further validated in large numbers of clinical samples. Second, a large prospective investigation and more in vivo and in vitro experimental studies are required to corroborate our results. Finally, the miRNA‒mRNA-TF network lacked experimental validation.

## Conclusions

The present study established a lipid metabolism-related diagnostic signature, which may have important diagnostic value for PCa patients. The TF-miRNA‒mRNA network may provide new diagnostic and therapeutic targets for PCa.

## Supplementary Information

**Additional file 1:** **Supplementary Figure 1.** U6 expression levels at the tissue and cellular levels. A Analysis of the expression levels of U6 in PCa (*n*=10) and BPH (*n*=10) tissue samples. B Analysis of the expression levels of U6 in PCa (DU145 and 22Rv1) cells and non-tumorigenic prostate epithelial (RWPE-1) cells. **Supplementary Table 1.** Receiver operating characteristic analysis for 27 differentially expressed lipid metabolism-related miRNAs.

## Data Availability

The raw data used in this study were extracted from the GEO database (https://www.ncbi.nlm.nih.gov/geo/query/acc.cgi?acc=GSE45604) and TCGA-PRAD database (https://portal.gdc.cancer.gov).

## Abbreviations

DEGs: Differentially expressed genes
qRT‒PCR: Quantitative real-time polymerase chain reaction
FC: Fold change
KEGG: Kyoto Encyclopedia of Genes and Genomes
ROC: Receiver operating characteristic; CI: confidence interval
SVM: Support vector machine
MSigDB: Molecular Signatures Database
SVM-RFE: Support vector machine recursive feature elimination
XGBoost: Extreme gradient boosting
KM: Kaplan‒Meier
BCR: Biochemical recurrence-free survival
RT: Reverse transcribe
OS: Overall survival
GEO: Gene Expression Omnibus
LMRMs: Lipid metabolism-related miRNAs
DEMs: Differentially expressed miRNAs
PSA: Prostate-specific antigen
TCGA-PRAD: The Cancer Genome Atlas Prostate Adenocarcinoma
LMRGs: Lipid metabolism-related genes
PCa: Prostate cancer
miRNAs: MicroRNAs
DE-LMRGs: Differentially expressed lipid metabolism-related genes
DE-LMRMs: Differentially expressed lipid metabolism-related miRNAs
BPH: Benign prostate hyperplasia
LASSO: Least absolute shrinkage and selection operator
TF: Transcription factor
AUC: Area under the ROC curve
NAFLD: Nonalcoholic fatty liver disease
