# Supplementary material for: Lipid metabolism-related miRNAs with potential diagnostic roles in prostate cancer
Source: Lipids Health Dis. 2023 Mar 14;22:39. doi: 10.1186/s12944-023-01804-4 (PMC10012590; doi:10.1186/s12944-023-01804-4)
Supplement: Supplementary file 1 — Additional file 1: Supplementary Figure 1. U6 expression levels at the tissue and cellular levels. A Analysis of the expression levels of U6 in PCa (n=10) and BPH (n=10) tissue samples. B Analysis of the expression levels of U6 in PCa (DU145 and 22Rv1) cells and non-tumorigenic prostate epithelial (RWPE-1) cells. Supplementary Table 1. Receiver operating characteristic analysis for 27 differentially expressed lipid metabolism-related miRNAs. [file 12944_2023_1804_MOESM1_ESM.docx]

**Supplementary Figure 1** U6 expression levels at the tissue and cellular levels. A Analysis of the expression levels of U6 in PCa (n=10) and BPH (n=10) tissue samples. B Analysis of the expression levels of U6 in PCa (DU145 and 22Rv1) cells and non-tumorigenic prostate epithelial (RWPE-1) cells.

**
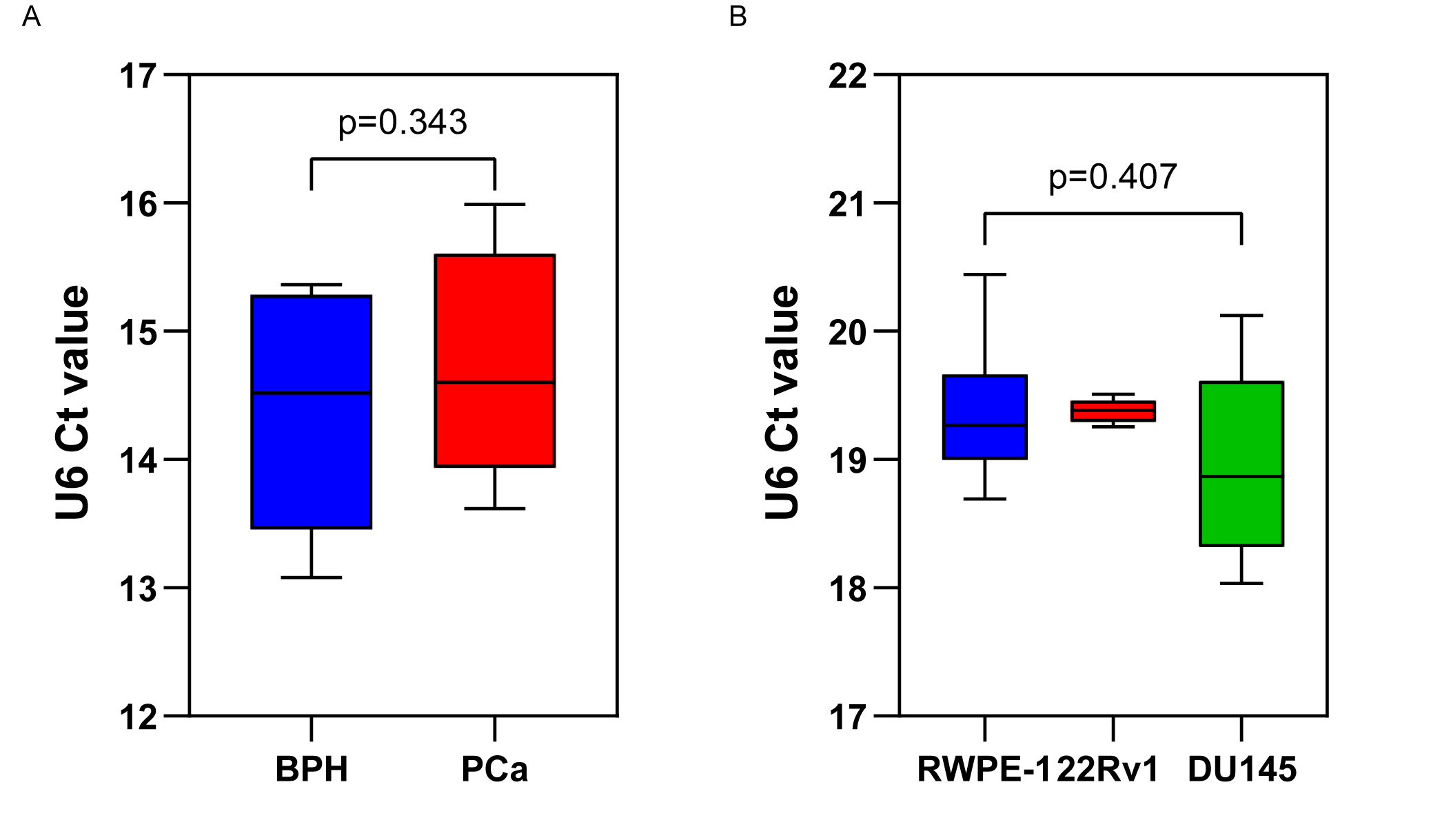
**

**Supplementary Table 1** Receiver operating characteristic analysis for 27 differentially expressed lipid metabolism-related miRNAs

| **miRNAs** | **AUC** | **95%CI** |
| --- | --- | --- |
| miR-93-3p | 0.97 | 0.955-0.986 |
| miR-182-5p | 0.96 | 0.923-0.989 |
| miR-200c-3p | 0.96 | 0.946-0.980 |
| miR-25-3p | 0.96 | 0.924-0.994 |
| miR-375-3p | 0.96 | 0.936-0.990 |
| miR-183-5p | 0.94 | 0.903-0.986 |
| miR-20a-5p | 0.94 | 0.922-0.967 |
| miR-96-5p | 0.94 | 0.913-0.973 |
| miR-148a-3p | 0.93 | 0.888-0.977 |
| miR-17-5p | 0.93 | 0.907-0.957 |
| miR-708-5p | 0.93 | 0.908-0.962 |
| miR-191-5p | 0.92 | 0.888-0.952 |
| miR-3074-3p | 0.92 | 0.890-0.944 |
| miR-425-5p | 0.91 | 0.862-0.953 |
| miR-106a-5p | 0.90 | 0.800-0.900 |
| miR-141-3p | 0.90 | 0.861-0.947 |
| miR-21-5p | 0.89 | 0.848-0.926 |
| miR-629-5p | 0.88 | 0.843-0.920 |
| miR-126-3p | 0.86 | 0.816-0.900 |
| miR-221-3p | 0.86 | 0.806-0.915 |
| miR-30b-5p | 0.84 | 0.782-0.893 |
| miR-20b-5p | 0.83 | 0.775-0.883 |
| miR-660-5p | 0.83 | 0.785-0.883 |
| miR-187-3p | 0.82 | 0.759-0.885 |
| miR-19a-3p | 0.82 | 0.772-0.866 |
| miR-363-3p | 0.78 | 0.719-0.846 |
| miR-210-3p | 0.70 | 0.633-0.773 |

*Abbreviation: CI* confidence interval
